# An emulation model in critical thinking used to develop learning outcomes in inter professional practice

**DOI:** 10.1002/cre2.195

**Published:** 2019-05-27

**Authors:** Kecia S. Leary, Leonardo Marchini, Jennifer Hartshorn, David C. Johnsen

**Affiliations:** ^1^ University of Iowa College of Dentistry & Dental Clinics Iowa City Iowa

**Keywords:** dental education, health care team, interdisciplinary health team, interprofessional communication

## Abstract

**Abstract:**

Interprofessional education (IPE) and interprofessional practice (IPP) are essential for the comprehensive care of patients. A goal of this paper is to articulate learning outcomes likely to improve patient outcomes. Yet learning outcomes in IPE are “systematically lacking” in consistency.

**Objective:**

An approach offered here and the main purpose of this paper is to develop and implement an IPE learning outcome by applying emulation concepts from the education literature. In dental situations, emulation has been used to derive the thought process of the expert succinctly enough for the novice to apply to the next patient.

**Methods:**

The expert's thought process thus becomes the learning outcome, the learning guide, and the assessment instrument. In IPE/IPP, several experts make up the team. The resulting learning outcome is the collection of key questions from respective health care team members. Team members are primary care, pharmacy, nursing, social work, nutrition, and dentistry. The resulting list of questions has not been reported and was applied to patient planning in a geriatric/special needs clinic.

**Results:**

Students were more likely to apply questions from disciplines that were preceded by didactic instruction—primary care, pharmacy, nutrition, and dentistry—and less likely to apply questions from nursing and social work.

**Conclusions:**

Although still in the early stages, the model is viable to guide learning and assess performance to a level of grasping the concept. The exercise is student led. For the practitioner, the learning outcome becomes the performance outcome. Further model development is ongoing with limited models for comparison.

## 1 INTRODUCTION

### 1.1 The international focus on interprofessional education and practice

Interprofessional education and practice (IPE/IPP) have been endorsed by every major academic health professions group. The current definition for oral health developed by the Federation Dentaire Internationale allows other health team members to understand how oral health is important for the patients' overall well‐being (Glick et al., 2016). Much progress has been made to enhance the culture of IPP. Extensive national efforts have been made in the United States to get health care providers together to better coordinate care so that outcomes are improved. However, gaps still remain in realizing a sustainable model for care coordination (D'Amour, Ferrada‐Videla, San Martin Rodriguez, & Beaulieu, 2005; Interprofessional_Education_Collaborative, 2016). Despite these efforts, the difference between IPP competencies and learning outcomes has not been clearly articulated, leaving a vacuum on what the student/practitioner is to do when interfacing with a patient, leaving a “wholesale lack of consistency” in articulating learning outcomes (Thistlethwaite & Moran, 2010). A major focus by academic health organizations have centered on IPE/IPP competencies. In 2016, the Interprofessional Education Collaborative published “Core Competencies for Interprofessional Collaborative Practice” (Interprofessional_Education_Collaborative, 2016).

### 1.2 The dilemma of establishing competencies before establishing learning outcomes and the wholesale lack of consistency for IPP learning outcomes

It is worth discussing and distinguishing competencies from learning outcomes. Definitions of competency have centered around capability, capacity, and competence. These perspectives are essential for gaining a larger view of capability, but they fall short of articulating what the practitioner is to do when interacting with the next patient. A literature review and synthesis by Thistlethwaite and Moran (2010) conducted on behalf of the World Health Organization discussed “The wholesale lack of consistency in defining and describing learning outcomes …” for IPP. An emerging concept in critical thinking centers on emulating the intended activity—what the student is to do (Johnsen, 2013; Johnsen, Lipp, Finkelstein, & Cunningham‐Ford, 2012; Lane & Stone, 2006). The emulation approach is consistent with approaches in learning outcomes. A widely accepted explicit definition of learning outcomes is elusive and summarized as follows: “The most commonly used and perhaps parsimonious definition of ‘learning outcomes’ proposes that they are ‘… what a student is expected to be able to DO as a result of a learning activity’” (Kraiger, Ford, & Salas, 1992). For health care practices, we currently lack a process that systematically includes the key perspectives of each member of the health care team regarding what each team member expects every other team member to do (or think) when encountering the next patient. In IPP, “do” means including each perspective into patient planning. A model is proposed for IPP consistent with the evolving definition of oral health being developed by Federation Dentaire Internationale where not only dental and oral conditions are considered. Physiological and psychosocial conditions are considered equally (Glick et al., 2016).

### 1.3 An emerging model suitable for IPP

An emerging approach in the methodology of critical thinking derives and describes the thought process of the expert succinctly enough for a novice to apply that process to a situation—in the health sciences, that can mean a patient or case (Benner, 2001; Johnsen, 2013; Lane & Stone, 2006; Marshall, Marchini, Cowen, et al., 2017). The approach is based on emulating the thought process of the expert, with the more direct the emulation, the greater the validity (Johnsen et al., 2012; Lane & Stone, 2006). The learning outcome is then expressed as the thought process of the expert(s). The thought process is also the learning guide and the assessment instrument. A challenge in IPP is that there is no single expert.

### 1.4 Purpose

The strategic purpose of the paper is to develop a learning guide to include the thought processes of health care team members for use in patient planning. The learning guide is also the educational/learning outcome and the assessment instrument. The enabling purposes of this project are to (1) apply an emulation model for student learning with learning outcomes in IPE/IPP based on (a) concepts from the Education literature previously applied to dentistry (Johnsen, 2013; Johnsen et al., 2012; Lane & Stone, 2006) and (b) recommendations by experts in eight IPP domains—primary care, pharmacy, nursing, nutrition, dentistry, social work, physical/occupational therapy, and home caregiver—to reach the threshold of systematic inclusion of each perspective into patient planning; (2) present the results for student performance following implementation of the learning exercise; and (3) discuss limitations and next steps.

For IPP, the ideal situation would be for health care team members to (a) have awareness of the cultures and general curricula of other health care members, (b) deliver care in the same location with the other team members, and (c) systematically apply explicit key perspectives from each team member for every patient. The first is being done with current IPE/IPP programs. The second is not practical on a prolonged national scale with practitioners that are geographically separated (for example, in rural areas). Iowa is largely a rural state. For relatively healthy people presenting to a single health provider with a specific request, formal input from all health team members may be unwarranted or impractical. The third is practical, has not been reported, and is the focus of this paper.

### 1.5 Context

As background for the students in IPE/IPP curricular initiatives, health science students in medicine, dentistry, nursing, pharmacy, physical therapy, health care management, and so forth begin in year one to learn the goals and curricula of fellow health professionals. Students engage in group activities such as exchanging the backgrounds and curricula of fellow students, working on team projects such as building pyramids, and engaging in case discussions. Pharmacy students perform a rotation in the College of Dentistry. Dental students have courses in physical assessment and review health histories for every patient in clinical care. Dental students also have courses in pharmacy and nutrition. A social worker was introduced in the college in 2017 and a nurse practitioner was added to the Geriatrics and Special Needs Clinic in 2016. Dental students did not have formal courses in nursing or social work but began working with these individuals in team approach to care. Before introducing the skillset with IPP Perspectives of Each Health Care Team Member, students failed to systematically apply each perspective.

A central theme is to develop a model with learning outcomes to guide IPP learning for the practitioner–patient interactions. It is assumed that development of a model will precede definitive assessment methodology. Thus, a more extended purpose is to offer an IPP learning model where the field is largely lacking and invite input and commentary on alternative models for more definitive study. Because learning outcomes are largely lacking for IPP, it is logical that definitive competencies will be refined with development of definitive learning outcomes.

## 2 METHODS

### 2.1 Concepts for learning guidance

The emulation concept of using the expert's thought process as the learning guide and assessment instrument follows general concepts of critical thinking and the “Novice to Expert” learning spectrum (Benner, 2001; Johnsen, 2013; Lane & Stone, 2006; Marshall et al., 2017). A challenge for IPP is that there are several experts. To overcome this challenge, a synthesis of the thought processes of the experts in various disciplines was derived and described succinctly enough for application to a patient or case. To develop a model skillset, the most important perspectives of each team member are derived and presented in Table 1. In developing an initial IPP learning model, the assumption was made that the team member with some years of experience in the IPE/IPP program has an adequate level of expertise for an initial model framework. Subsequent refinements can include validation of the most important questions from team members.

**Table 1:** Interprofessional skillset

Interprofessional skillset	
Provider	Question to ask
Patient	Preferences and expectations
Primary care provider	Prioritization of condition(s) that is/are life threatening or affect health
Pharmacy	Patient problems that are (or are potentially) drug related
Nursing (RN/LPN)	Patient capacity (factoring burden of conditions) to subscribe to treatment recommendations
Dentistry	Dental conditions/risk factors that affect (or are indicators of) general health Preventive measures for oral health based on overall health
Nutrition	Nutritional factors contributing to disease/condition, asking what the patient eats and drinks on a daily basis
Social worker/ counselor/behavioral health specialist	Barriers/solutions based on the home situation (money, transportation, organization, availability of assistance, etc.) Who is able to consent for this patient
Physical therapist/occupational therapist	Long‐term outcomes for therapy programs
Family caregiver	Person(s) responsible for the patient's daily living activities (consent, finances, etc.)
Patient	Assent

Members of the health care team were interviewed and asked “What is/are the most important perspective(s) you recommend every other member of the team incorporate when planning the next patient?” Interviewees were from primary care, pharmacy, nursing, social work, nutrition, dentistry, physical/occupational therapy, and a home caregiver. Primary care is defined as health care at a basic rather than specialized level for people making an initial approach to a doctor or nurse for treatment. All had been involved in the IPE/IPP program. A succinct skillset resulted (Table 1). For example, the pharmacist recommends for every member of the team to determine/consider which of the patient's health problems are drug related. The nurse recommends for every member of the team to determine/consider the capacity of the patient to subscribe to the treatment plans recommended, and so on for various disciplines. This approach may seem self‐evident but has not been explicitly applied to IPP. Although the questions derived from respective team members reflect expertise, the resulting list of questions is largely common sense. The authors are not aware of a comparable set of questions being published previously.

Each of the health perspectives is presented in a condensed format (Table 1). The skillset is designed to elicit follow‐up questions once an alert has been raised in a specific area. For example, for pharmacy, the initial perspective is “Which of the patient's problems are drug related?” If there are drug‐related problems, follow‐up questions can be asked about drug actions, side effects, common drug interactions, wrong drug, wrong dose, and so forth.

### 2.2 Logistics

Program participants were 69 fourth‐year dental students who took part in a 5‐week Geriatrics and Special Needs Clinic rotation in 2016, 2017, and 2018. Iterations of the learning exercise were introduced and refined for 2 years before the data presented here were collected. Students were introduced to the IPP concept of systematically including the perspectives of each health care team member during their orientation to the rotation. This study was granted exempt status by the University of Iowa Institutional Review Board # 201512721.

During their rotations, fourth‐year dental students were asked to select a patient whom they cared for during their time in the rotation for a case presentation in which they followed the steps in the combined geriatric risk‐IPP learning guide. Faculty offered guidance for case selection and documentation. A PowerPoint template was provided (based on previous comments from the students) to enhance student presentations. Students presented their cases to faculty members and peers in a 20‐min time frame (10‐min presentation and 10‐min discussion). Six patients were presented in each session.

### 2.3 Assessment

**Figure 1:**
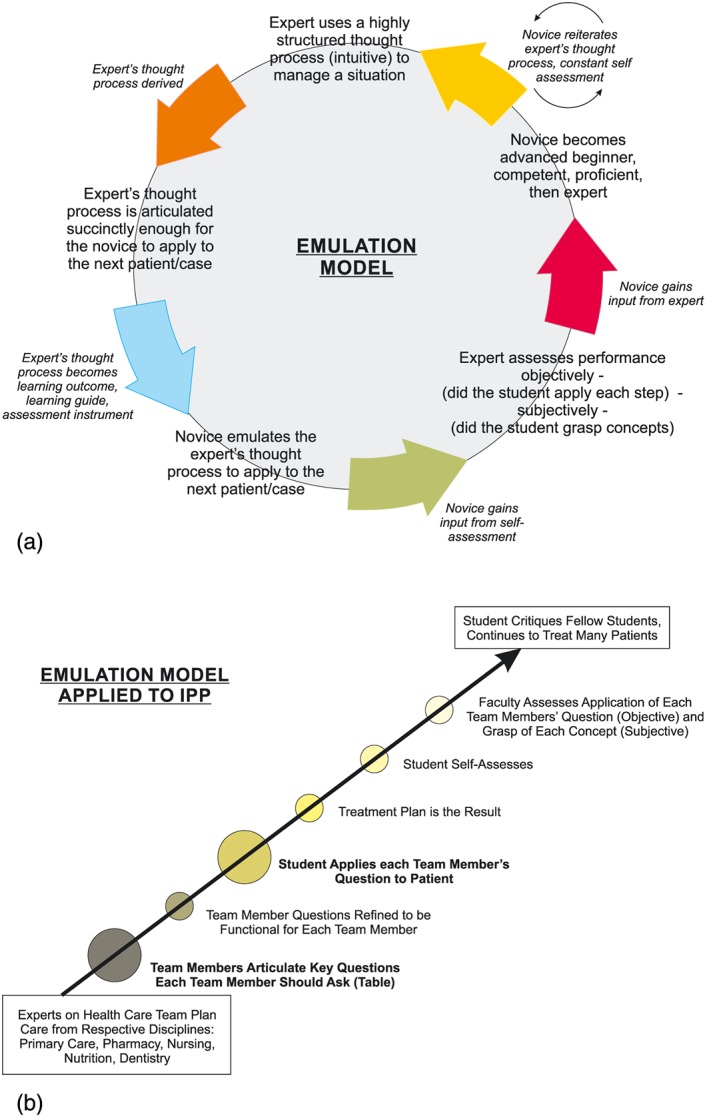
(a) Schematic for an emulation model as the basis for skillsets in critical thinking in dental situations including treatment planning, literature search and critique, caries risk, geriatric risk, evidence‐based dentistry, and for this paper, interprofessional practice (see the application of this emulation model to IPP in Figure 1b). (b) Schematic for application of this emulation model to interprofessional practice. Each step of the emulation model is applied to the next patient starting with deriving the thought processes of the experts (the health care team) succinctly enough for the novice to apply to the next patient

One faculty member (Examiner 1) evaluated the performance of all the students, with a second assessment for 22 of the presentations. Learning outcomes in this context means integrating the multi‐health team framework into patient/case‐based presentations (Figure 1). The assessment threshold means including the thinking of each team expert into patient planning. Although not an explicit purpose, the project was expected to offer feedback on how successful the penetration of different health perspectives was when moving from didactic courses to the minds of students during the formation of patient care plans. Students progressed rapidly through the Perspectives of Each Health Care Team Member exercise. As with previous critical thinking exercises, performance was based on demonstrating a systematic thought process that resulted in alternative treatment options rather than merely getting the “right” answers. Thus, the learning guide served as the assessment instrument. Each health team perspective is in the PowerPoint template. Thus each student at least minimally alludes to each of the perspectives. At this stage of model development, assessment is limited to whether the student goes beyond reading the perspective to incorporate the health perspective into patient planning.

## 3 RESULTS

Students nearly all the time included some mention of each discipline because each was on the learning guide. As an initial effort to gage grasp of the concept of each question, faculty assessed the grasp of the response to the question (Table 2). Random corroborating assessments were done. The assessment of “grasp of concept” is the focus for further refinement. A key assumption is that a practitioner repeatedly asking the right question will get better at answering the question with experience. Implementation of the learning guide with the perspectives of each health team member was successful in that each student completed the presentation and considered each step. A surprising finding was the spectrum of penetration of health perspectives as will be expanded in Section 4.

**Table 2:** Number and percentage of times team member perspective was incorporated by the students into patient planning (n = 69)

Team member	Number and percentage of times team member perspective was incorporated by the students into patient planning (n = 69)
Primary care provider	57 (82.6%)
Pharmacy	50 (72.4%)
Nutrition	46 (66.6%)
Social work	37 (53.6%)
Nursing	35 (50.7%)

One design feature of the exercise is the frequency of incorporating health perspectives into patient analysis. The result is that each student systematically incorporates eight health perspectives for their patient. In addition, each student observes five other students incorporating eight health perspectives for a total of 40 health perspectives being systematically incorporated into patient care in one session. The potential importance of the environment for repetitive and extensive exposure to the systematic approach will be in Section 4.

To analyze responses, 69 patients were presented by students in the Geriatrics Special Needs Clinic with each student expected to systematically incorporate the perspectives of eight health team members for each patient. Table 2 shows the number of times the students incorporated respective perspectives of that team member into patient planning.

The primary care perspective was most often included. The difference between primary care and nutrition was statistically significant (*X* ^2^ = 4.12; *p* = .042) There was no difference in the occurrence of students including the pharmacy and nutrition perspectives (*X* ^2^ = 0.38; *p* = .54; NS). Students were more likely to include the pharmacy perspective than the nursing perspective into patient planning, and the difference was statistically significant (*X* ^2^ = 6.98, *p* = .008). As the course progressed, improvement was seen in the inclusion of the nursing and social work perspectives. Comparing the first 33 patients with the next 17 patients, students were more likely to include the social work perspective for the next 17 patients than for the first 33 patients (*X* ^2^ = 6.18; *p* = .012). Students were more likely to include the nursing perspective for the next 17 patients than for the first 33 patients, but the difference was not significant (*X* ^2^ = 2.88, *p* = .089). It is noted that pharmacy and nutrition have been formal courses in the college for decades. Nursing and social work have not had courses, but providers were added to the clinic in 2016. Although not an explicit purpose, the project was expected to offer feedback on how successful the penetration of different health perspectives was when moving from didactic courses to the minds of students during the formation of patient care plans.

Calibration was performed by two dental faculty members. For performance assessment calibration of examiners, 22 patients were presented by students, with eight perspectives for each patient/case for a total of 176 possible points of agreement/disagreement. Agreement occurred for 164 of the 176 points (93%).

## 4 DISCUSSION

### 4.1 The emulation model

Although this is not the definitive model in establishing learning outcomes for the health care team in IPP, it is a step forward. We are not aware of a systematic collection of key perspectives for the health care team articulating what every member of the team is to do (think) in their next patient encounter. We submit that systematic inclusion of each team member's thought process enhances the systematic improvement of planning and risk assessment for such patients. We further submit that this model is educationally sound and practical in guiding learning for team members. Although the emulation model applied to IPP was used by dental students, this skillset is practical for any member of the health care team.

### 4.2 Student performance and the case for didactic instruction in all disciplines

Penetration level, or lack thereof, for the perspectives of health care providers became clear in a way that may not have been possible without an explicit skillset. The association suggests that students with formal instruction in a discipline are more likely to include the thinking and health perspectives into patient care. It is noted that a full‐time social worker had been included in the fall of 2017 and may be a factor in improved inclusion of that perspective. The perspectives with formal coursework in the students' curriculum were the ones more often included during this exercise. The association between the extent of inclusion for a health perspective and the extent of didactic curriculum experience may be a key finding. This association can lead to increasing didactic instruction as part of a progression to the clinic, as with previously reported critical thinking exercises(Guzman‐Armstrong, Warren, Cunningham‐Ford, von Bergmann, & Johnsen, 2014). Nursing was judged by faculty to be the weakest—“What is the patient's capacity to subscribe to recommended treatments?”

As with previous critical thinking exercises, this one took multiple years to make it feasible and clinically relevant (Marshall et al., 2017; Figure 1/schematic). As with previous critical thinking skillsets, the IPP skillset saw improvement over presentations that had no skillset as a learning guide (Marshall et al., 2017). The inclusion of the IPP skillset into the geriatric risk skillset seemed logical because the majority of patients in this clinic are affected by problems relevant to the domains of multiple health care workers (Marchini, Hartshorn, Cowen, Dawson, & Johnsen, 2017). Health issues in this clinic include dementia, stroke, Parkinson's disease, and multiple medications with side effects that result in dry mouth, mental health issues, cancer treatment, organ transplants, diabetes, and trauma, just to give examples of the vast diagnoses of patients.

The significantly higher penetration of some perspectives is interpreted as a reinforcement of the importance of formally including these perspectives in the didactic part of the students' curriculum. For primary care, it seems logical that the course in physical assessment plus the daily review of each patient's health history will be associated with a more systematic inclusion of this perspective. The course in pharmacy plus the presence of two full‐time pharmacists, as well as the health history question on medications, reinforces the impact of these formal curriculum elements on the frequency of including the pharmacy perspective into patient planning. The same holds for nutrition; a course in nutrition and a full‐time nutritionist on faculty are associated with greater penetration for nutrition than for disciplines without didactic instruction. With the addition of a social worker, the penetration improved and will likely continue to improve as students have more exposure to this perspective on a day‐to‐day basis. The notion of engaging multiple health care disciplines as a means for a more in‐depth view of patient care is reinforced with this project (Brandt, Lutfiyya, King, & Chioreso, 2014; Gauger, Prosser, Fontana, & Polverini, 2018; Harnagea et al., 2017; O'Malley & Reschovsky, 2011).

### 4.3 An evolving model and limitations

Assessment is in the early stages of development. The threshold of inclusion into patient planning seems adequate for this stage of development—including demonstrating when a health perspective does not need to be used in patient planning. Continued adjustments are planned for calibration and to ensure consistency in assessment. Future directions can also include the addition of perspectives for other team members, for example, the behavioral therapist.

The skillset has limitations. An inherent limitation is that every critical thinking skillset is incomplete and in a constant state of revision. Limitation of assessment to dental faculty excludes in‐depth assessment by fellow team members. The next step is to include a fellow team member (a social worker) in the assessment process, which will likely increase the number of disagreements among those who evaluate the student presentations. The current model does not have a formal remediation protocol. Although the geriatric clinic setting makes for a rich IPP experience, complicated patients rarely have clear treatment plans. These complicated patients can have several alternatives, making clear performance assessments by faculty equally challenging (Marchini et al., 2017).

## 5 CONCLUSION

The emulation approach considered here offers a succinct and systematic way to integrate the perspectives of each health team member into learning outcomes. The focus is on learning and assessment using the same instrument. The emulation approach applied here follows educational concepts previously reported. The exercise was successful in getting each student to consider the key perspectives of each health team member. The emulation approach revealed strengths and weaknesses of the curricula for each of the health perspectives. Specifically, primary care, pharmacy, and nutrition were stronger—where didactic instruction was long‐standing—than were nursing and social work, where didactic instruction was minimal. A student has met the assessment threshold when the student incorporates the perspective into patient planning—or demonstrates that the perspective will not change planning. Calibration efforts showed further refinements are needed to assess the depth of the student's grasp of each health perspective. The learning framework builds on previous work challenging the student to use judgment to coincide with the thought processes of the health team members. In so doing, the model expands the body of scholarship in the critical thinking domain.

## CONFLICT OF INTEREST

All authors declare there are no conflicts of interest in relation to this study.
